# Role of saltmarsh systems in estuarine trapping of microplastics

**DOI:** 10.1038/s41598-022-18881-7

**Published:** 2022-09-15

**Authors:** Chiedozie C. Ogbuagu, Hachem Kassem, Udiba U. Udiba, Jessica L. Stead, Andrew B. Cundy

**Affiliations:** 1 Department of Geology, Faculty of Physical Sciences, University of Nigeria, Nsukka, 410001 Nigeria; 2 School of Ocean and Earth Science, National Oceanography Centre (Southampton), University of Southampton, Southampton, SO14 3ZH UK; 3 Department of Zoology and Environmental Biology, University of Calabar, Calabar, 540271 Nigeria; 4 Southern Marine Science and Engineering Guangdong Laboratory (Guangzhou), Guangzhou, China

**Keywords:** Geochemistry, Sedimentology, Ecology, Zoology, Environmental sciences, Ocean sciences, Solid Earth sciences

## Abstract

Saltmarshes are important natural ecosystems along many temperate (and other) coastlines. They stabilize sediments and act as biofilters for a range of industrial pollutants and, potentially, microplastics. Accumulation of microplastics along estuarine coastlines may be enhanced by the presence of saltmarsh species, as they offer better particle trapping efficiency than adjacent intertidal mudflats under prevailing flood and ebb tidal currents. However, the trapping efficiency of entire saltmarsh systems under varying flow conditions has not been widely assessed. While the effects of saltmarsh systems on water flow, and on sediment transport and trapping, have been relatively well studied, little is known about the contributions of saltmarsh halophytes, resident organisms and the associated saltmarsh sediments to the trapping of microplastics. To address this, a series of flume experiments were undertaken to examine transport and accumulation of Bakelite particles (~ 500 µm) and PVC nurdles (~ 5 mm) as model plastics in sub-sampled saltmarsh and intertidal mudflat monoliths. The results showed that saltmarsh systems influenced the hydrodynamics within and above the canopy, enhancing turbulence and shear stresses. With increasing flow velocities (≤ 0.51 m s^−1^), negligible quantities (2 $\times$ 10^−4^ mg L^−1^) of sediments and Bakelite particles were eroded and resuspended. The algal biogenic roughness from the mudflat, and the vegetative roughness from the *Spartina* plants on the saltmarsh, inhibited the transportation of the microplastics within the tested systems. Resident burrowing crabs (*Carcinus maenas*) promoted the burial, release and transport of microplastics. The results of this study provide evidence of the contributory roles of saltmarsh systems in the sequestration of microplastics and sediment stabilization. Estuarine saltmarsh systems can act as sinks for microplastics with enhanced burial from burrowing crabs under favourable flow conditions.

## Introduction

Vegetated intertidal habitats provide diverse support to natural, coastal and estuarine systems and valuable ecosystem services including protection against coastal hazards, regulation of contaminants and pathogens, and provision of habitats for macrobenthos such as bivalves, fishes, gastropods, crustaceans, and other invertebrates^1–4^. The positive contribution of coastal vegetated habitats to shoreline protection^5^ is partly attributed to the combined inputs of the flora and fauna which occupy these habitats. The flora provide an enhanced biogeneic roughness which reduces the hydrodynamic shear stress^6–8^ thus decreasing the erosion rate and prompting accumulation/deposition of sediments. Meanwhile, the fauna offer biostabilization of the sediments through the secretion of biopolymers^9^, generation of armoured surfaces and other mechanisms that minimize the risk of erosion^10,11^. Mangroves, saltmarshes, seagrasses and reed beds occupy most estuarine coastlines that provide these biomorphodynamic and bioengineering functions^3,12,13^. Benthic organisms may also induce bioturbation, weakening the sediment bed through their grazing and borrowing activities.

Recent studies have described saltmarshes to be more efficient in stabilizing sediments along estuaries than other wetland types^3,14^. Cordgrasses within saltmarshes (for example, the *Spartina* plant species of *Spartina alterniflora* and *Spartina anglica*) provide appreciable influence on the ability of saltmarshes to reduce tidal current velocities when fully submerged at high tide and hence reduce sediment transport^8,15–17^. Neumeier and Ciavola^18^ described how the structure of the *Spartina* plants minimizes sediment transport, whereby stems and leaves induce turbulence, vary near-bed shear stresses and alter the hydrodynamics overlying the saltmarsh beds^7,17^. In addition to sediment trapping and stabilization, the efficiency of *Spartina* saltmarshes in trapping other particles such as macro- and microplastics was reported by Yao et al*.*^19^.

Macro- and microplastics trapping or accumulation in estuarine saltmarshes were previously reported^14,19–23^ as part of an estuarine “filter” that can reduce the fluxes of river-derived plastics to coastal and open marine environments. The concentration of microplastics (defined as having a diameter less than 5 mm) in some estuaries may be lower than that of macro-plastics, but a higher concentration of the former is usually recorded with time as the macro-plastics disintegrate^17,19^. Like their sediment trapping and stabilization role, the estuarine saltmarsh system, which transitions from bare mudflat to fully vegetated saltmarsh beds, may favour a higher trapping efficiency of these microplastics than other wetland types^14,19^, preventing them from being transported offshore by ebb tides and onshore by the flood tides. This higher trapping efficiency stems from the structure of their halophytes, notably *Spartina* plants. The presence of stiff stems and long leaves in the canopy with the encrustation of green algae and other epiphytes^24^ at the lower parts enables *Spartina* plants to suppress tidal current velocities, trap microplastics and bind the sediments effectively. Additionally, higher bed elevation/topography of mudflat and saltmarsh beds may instigate the trapping (or release) of macro- and microplastics along estuaries at and around slack water periods. Regardless of the variations in the bed topography, bare mudflats show weaker trapping efficiency for both macro- and microplastics, and this is particularly apparent at higher flow velocity or storm conditions^19^.

**Figure 1:**
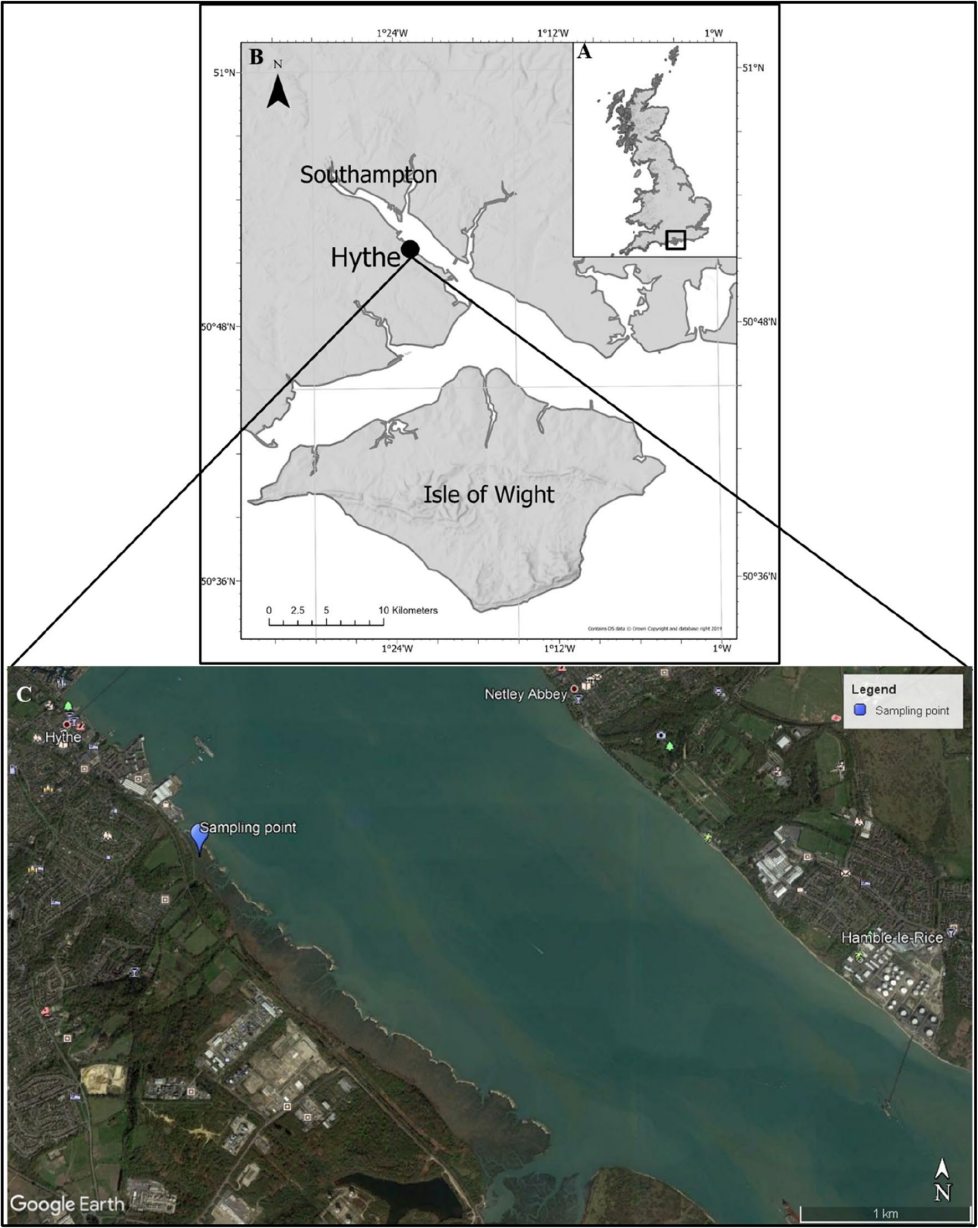
Hythe intertidal zone, Southampton Water, UK: sampling site for the saltmarsh systems examined in the present study. Aerial photographic imagery Copyright 2021 Google. Map data Copyright 2021 Google.

Several recent papers highlight the increased transport or flushing of plastics from sediment beds under storm or flow conditions beyond the threshold of motion, particularly in river systems^25,26^. Given the likelihood of frequent coastal storms and other high energy events along coasts and in estuaries, there is a need to investigate the microplastic trapping efficiency of estuarine saltmarsh systems at flow velocities higher than dominant flood and ebb tidal current velocities. Further, the influence of saltmarsh halophytes in the trapping of microplastics has been the focus of most published work, while the impact of sediment infauna in trapping, burial and remobilisation of microplastics has received little attention. The roles of burrowing crabs and other infauna within estuarine saltmarshes^9,27^ in the stabilization, bioturbation and reworking of cohesive sediments may also be influential in the microplastics trapping efficiency of saltmarshes. This research paper aims to assess the role of saltmarsh flora and fauna in microplastics trapping/release in temperate mudflat and saltmarsh systems under varying flow conditions, using saltmarsh systems from Southampton Water, United Kingdom, as an example (Fig. 1). Specifically, this paper addresses the following objectives: (1) to assess the effect of saltmarsh and mudflat beds on hydrodynamics at varying current velocities, (2) to compare the effect of saltmarsh and mudflat beds on the transport and trapping of two forms of microplastics and (3) to examine the role of bioturbators in trapping, burial, and release of microplastics.

## Results

### Hydrodynamics properties of flow in saltmarsh and mudflat

**Figure 2:**
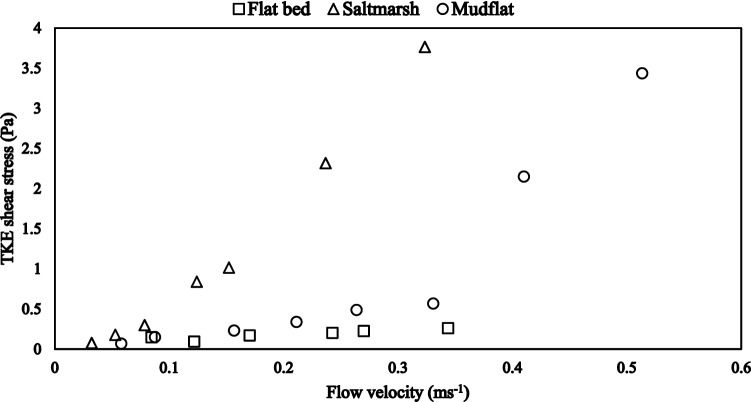
Mean flow velocity and TKE shear stress distributions for the three experimental runs; Test A (flatbed clear water), Test B (mudflat/flat sediment bed) and Test C (saltmarsh/vegetated sediment bed). Vertical distributions of the flow velocities and TKE shear stresses for the three experimental runs are presented in Figs. S1, S2 and S3.

Constant flow distribution of current velocities was recorded within annular flume experiments, across the profiles, for tests A and C (i.e. flatbed clear water and flat sediment bed; see “Experiment setup”). Experimental runs for Test A recorded lowest and highest mean velocities of 0.05 m s^−1^ and 0.39 m s^−1^, respectively. The Test C experiment recorded the highest mean flow velocity of 0.51 m s^−1^ at 45 Hz motor speed (Fig. 2). Flow velocity distributions within the vegetated sediment bed (Test B) were constant, up to 4 cm above the saltmarsh bed. Above this height, the flow velocities showed a typical logarithmic-shaped velocity profile (Fig S2). Tests B and C followed the quadratic bottom-stress law, with the increase in shear stress as a function of velocity showing a parabolic curve (Fig. 2).

Turbulent kinetic energy (TKE) shear stresses within tests A and C also showed a constant distribution across the vertical profiles (Figs. S1 and S3). Consequent to the derivation of TKE from velocity fluctuations, the TKE shear stress distribution for test B (vegetated sediment bed) was consistent with the test’s mean flow velocity distribution. However, of the three experimental runs, Test B recorded the maximum TKE shear stress of 3.4 Pa (Tables S1 and S2).

A turbulent flow regime characterized the flow states for the three experimental runs with Reynolds number (Re) of 1.2 $\times$ 10^5^, 1 $\times$ 10^5^ and 1.2 $\times$ 10^5^, for tests A, B and C, respectively.

### Sediment and microplastics transport

**Figure 3:**
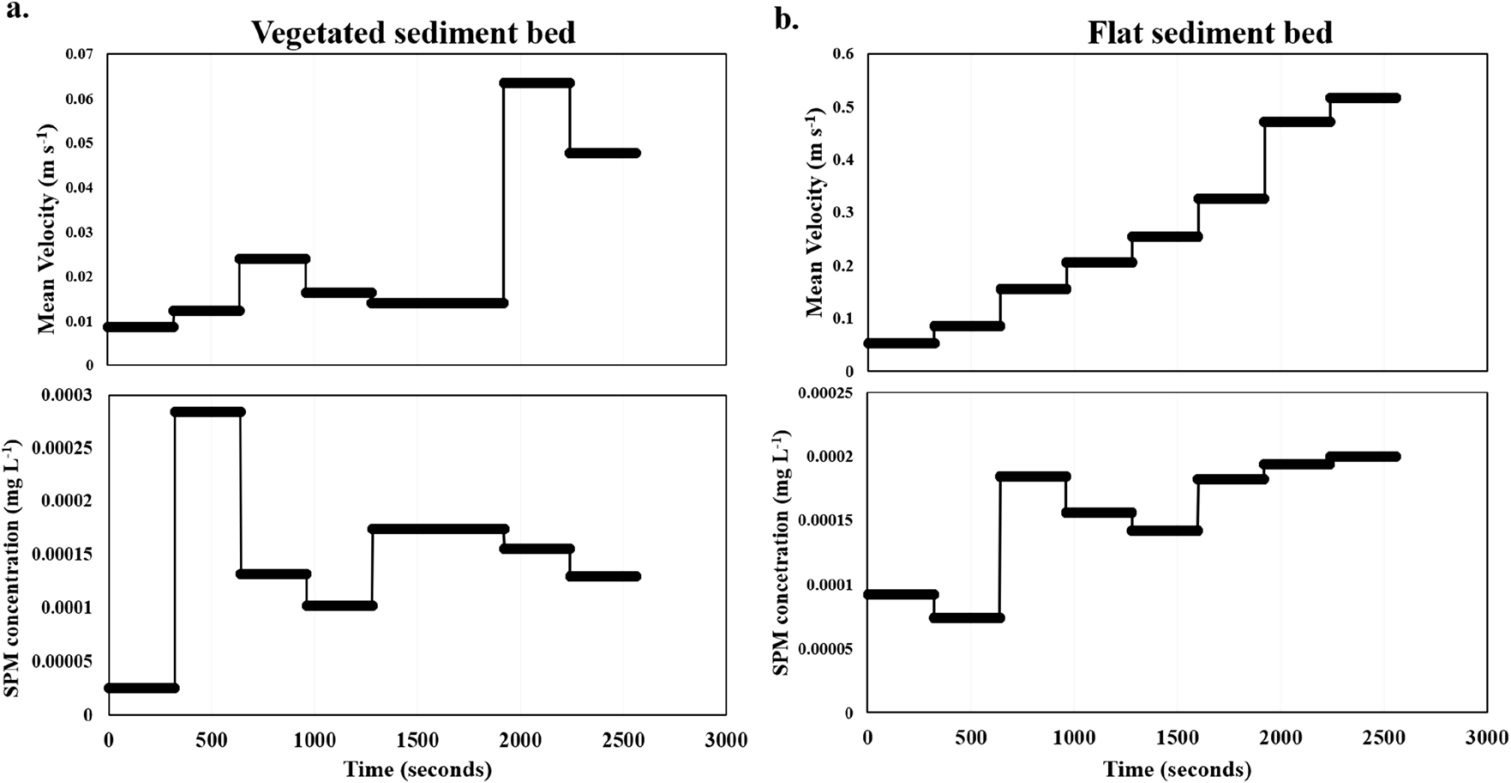
Time series plots of the suspended particulate matter (SPM) concentrations and mean velocity for (**a**) vegetated sediment bed and (**b**) flat sediment bed at a height of 3 cm above the bed (Profile 3).

Time series plots of suspended particulate matter (SPM) concentrations, derived from Optical Backscatter Sensor (OBS) readings and mean velocities showed some discrepancies between the OBS, SPM concentrations and the increasing velocities. The SPM concentration comprises the resuspended matter (sediment and organics) and microplastics. This complicated the calibration of OBS measurements with SPM concentrations (see “Hydrodynamics measurement, microplastics and sediment sampling methods”). However, the SPM concentrations and mean velocity time series for the vegetated sediment bed presented little or no changes (Fig. 3a). Mean velocity at the 3 cm height (used for the time series plot) remained below 0.03 m s^−1^ up till 40 Hz (0.063 m s^−1^). Residues from the filtered water samples comprised algal debris and countable Bakelite particles. Minimum and maximum SPM concentrations (including the Bakelite particles) for the vegetated sediment bed were between 10^–5^ mg L^−1^ and 10^–4^ mg L^−1^ and remained at approximately 1.5 $\times$ 10^–4^ mg L^−1^ for most velocity ranges.

The flat sediment bed recorded a significant increase in SPM concentrations with increasing mean flow velocities. Before attaining a mean flow velocity of 0.25 m s^−1^ (30 Hz motor speed), the SPM concentrations remained at background levels with increasing flow velocities (Fig. 3b). Higher flow velocities above 0.25 m s^−1^ caused a stepwise increase in the resulting SPM concentrations from 1.4 $\times$ 10^–4^ mg L^−1^ to 2 $\times$ 10^–4^ mg L^−1^. Similarly, the suspended material is generally composed of algal debris and Bakelite particles, as retained following filtration.

From visual records (see Supplementary Video S1), the PVC nurdles were mainly transported as bedload, with traction/surface creep and rolling at low velocities < 0.1 m s^−1^ and by saltation (rolling and jumping) at velocities higher than 0.26 m s^−1^, in both tests B and C (vegetated bed and flat sediment bed, respectively). At low flow velocities, the PVC nurdles made intermittent jumps and were halted by the algal biofilm mat. The estimated saltation (jumping) speed from visual observations was 0.003 m s^−1^. Bedload (traction and rolling) movements of the PVC nurdles were dominant in the vegetated sediment bed.

### Trapping and burial of microplastics

The trapping and burial efficiencies of the saltmarsh and mudflat monoliths were deduced from video recordings and syringe core analysis. Bakelite particles were observed to be incorporated within algal biofilms and debris forming aggregates on the mudflat. Subsequent erosion, disintegration and resuspension of the aggregates depended on the consequent flow velocity regime (≥ 0.25 m s^−1^) and burrowing/feeding activities of the resident crabs, C*arcinus maenas.* Analyses of the video records revealed that crawling of the crabs entrained the PVC nurdles, as well as mobilized/resuspended the Bakelite particles and top layer of fine sediments along their paths.

**Figure 4:**
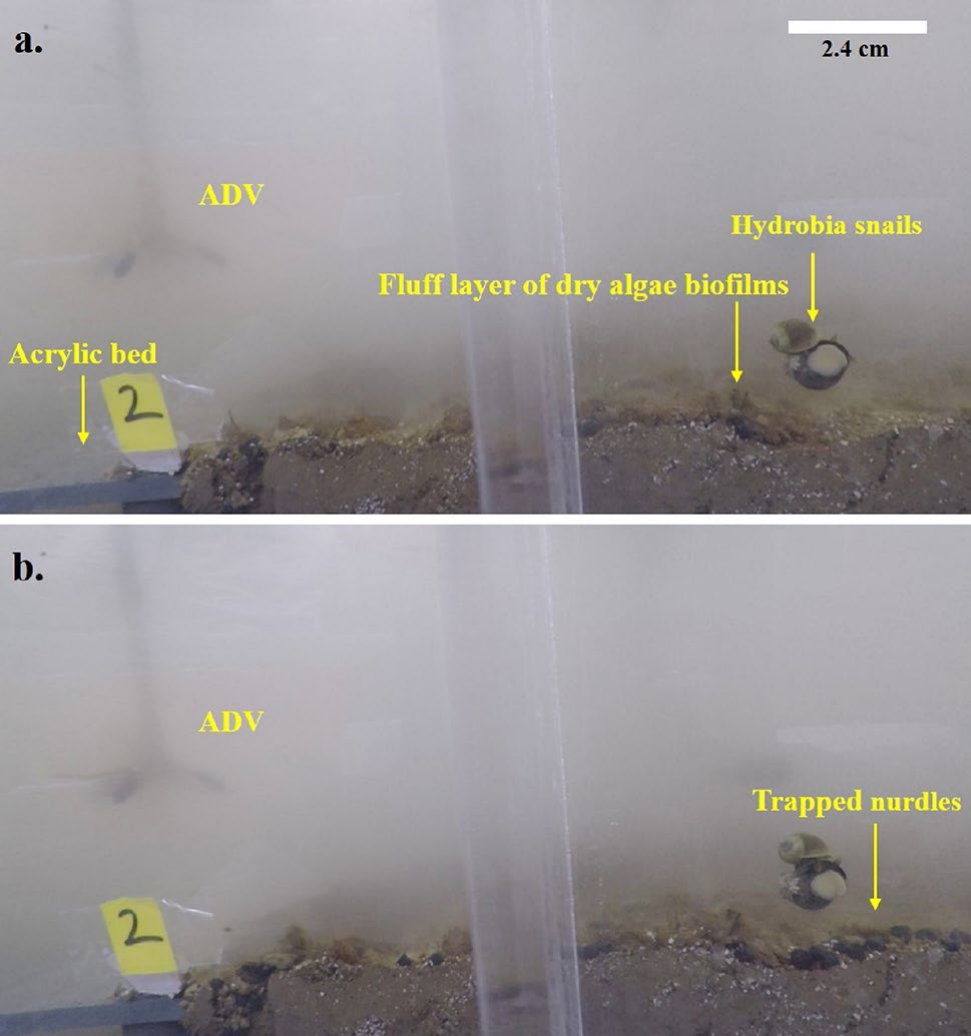
Trapping of the nurdles by the biogenic roughness from dry biofilm layer. Note the effect of depression on the bed surface in trapping the nurdles.

**Figure 5:**
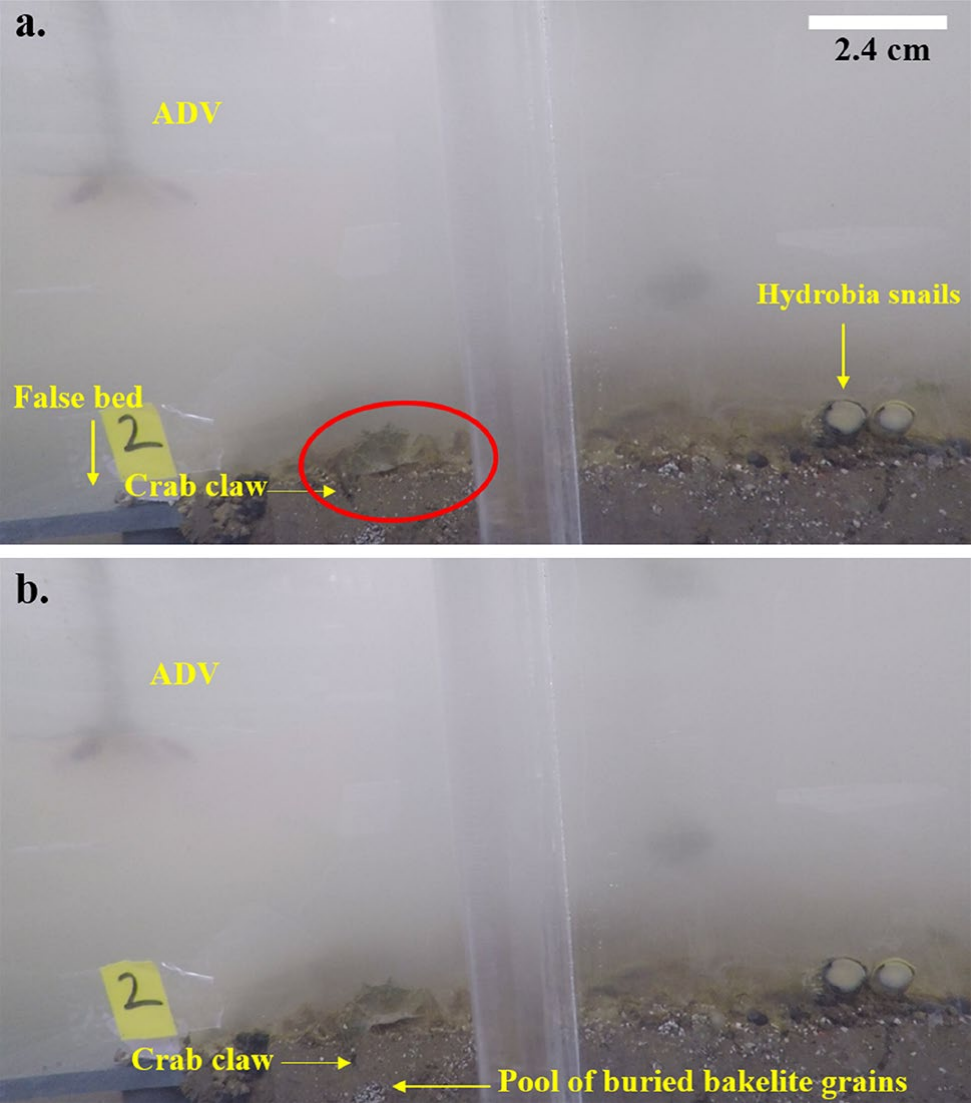
Burial of Bakelite particles by the burrowing crab *Carcinus maenas*. (**a**) Onset of the crab inserting its claw into the burrow it formed. (**b**) Extension of the claw to about 1 cm depth by the crab to reach the pool of Bakelite particles. This action continues repeatedly with particles sinking into the burrow (See Supplementary Video S2).

**Figure 6:**
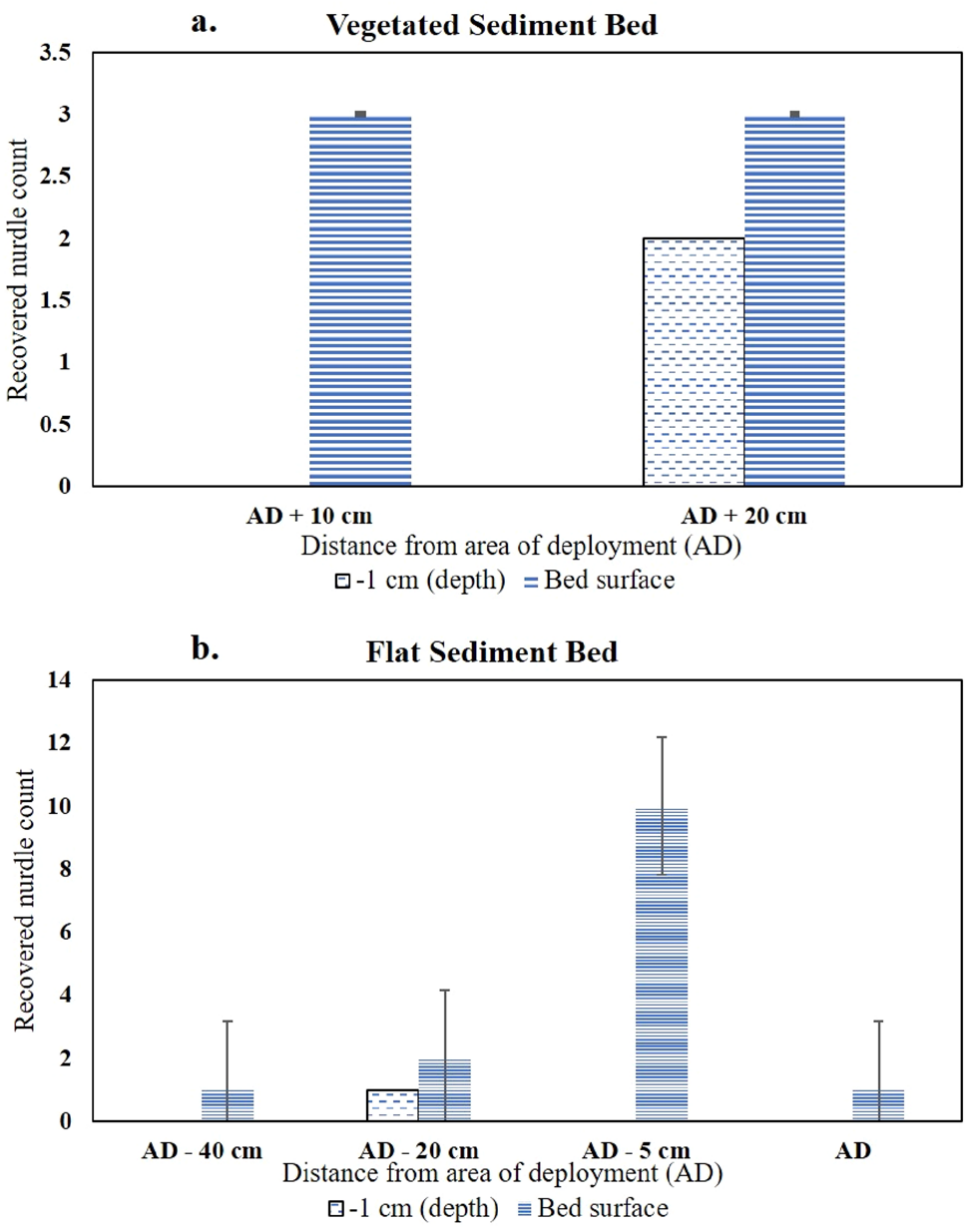
Number of nurdles recovered from post-experiment cores and their depths for (**a**) vegetated sediment bed (Test B) and (**b**) flat sediment bed (Test C). AD means Area of Deployment; the negative sign means upstream (against the water flow direction) and positive sign means downstream (in the direction of the water flow).

**Figure 7:**
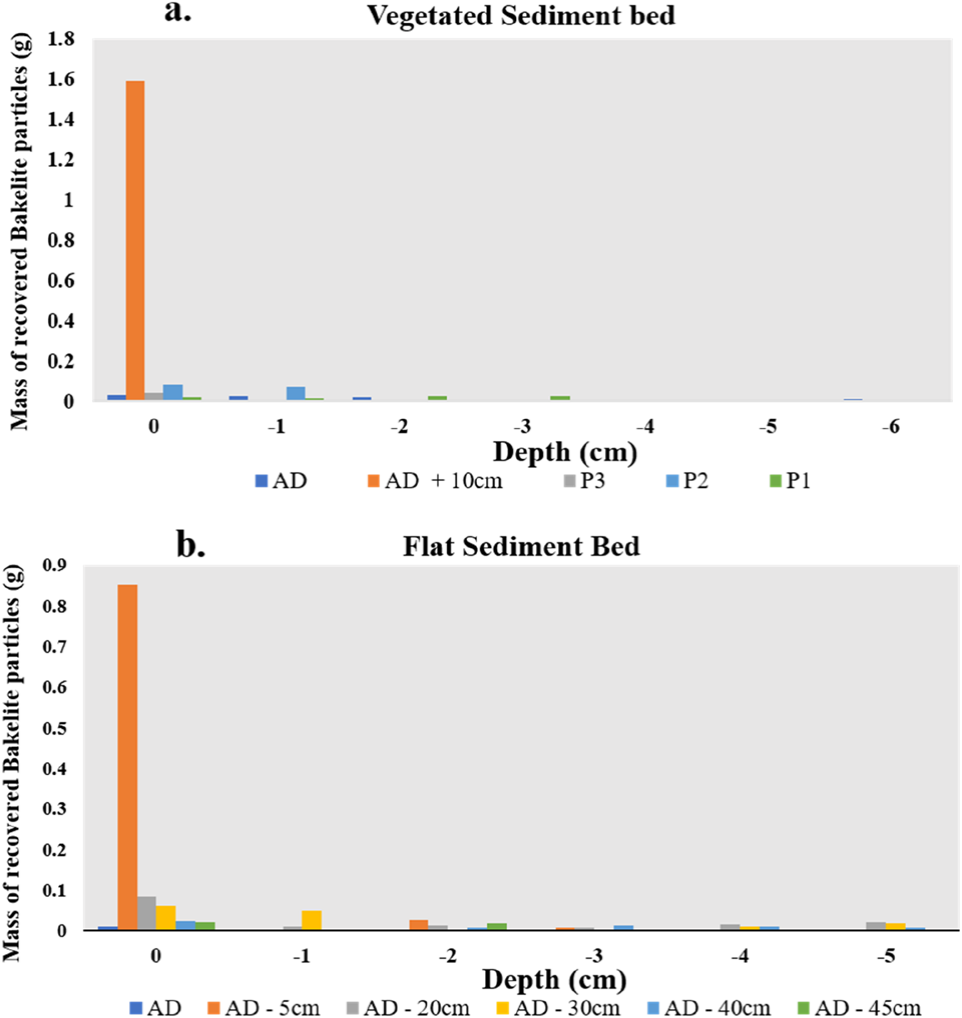
Mass of recovered Bakelite particles from post-experiment cores and their depths (in cm) for (**a**) vegetated sediment bed (Test A) and (**b**) flat sediment bed (Test C). AD means Area of Deployment; the negative sign means upstream (against the water flow direction) and positive sign means downstream (in the direction of the water flow).

Bed roughness (bed topography and roughness of biofilm, aggregates and pellets at the surface) of the mudflat facilitated the trapping of the PVC nurdles as they slid or rolled, while vegetation density and bed roughness played a similar role within the saltmarsh. Monitored sections of the flat sediment bed experiment showed accumulation of the PVC nurdles and Bakelite particles (Fig. 4) at flow velocities < 0.1 m s^−1^. The plastic particles are subsequently buried by the deposition of algal aggregates from the flow. An additional burial mode of microplastics observed was from the burrowing activity of crabs. Pools and individual grains of Bakelites occurring within the subsurface were observed to be buried by the grazing and burrowing activities of the crabs (Fig. 5). Recovered PVC nurdles from post-experimental syringe cores sampled from the saltmarsh and mudflat beds (Tests B and C, respectively) revealed a maximum burial depth of 1 cm (Fig. 6). Displacement of the PVC nurdles from the area of deployment (P3) extended up to 40 cm upstream in the mudflat, while they were displaced to a maximum of 20 cm downstream within the saltmarsh. Weighted mass calculations of buried Bakelite particles from the post-experimental syringe cores showed a higher concentration of the particles within 5 to 10 cm of the area of deployment. The maximum burial depth in the mudflat was 5 cm extending to 6 cm in the saltmarsh. Greater dispersal of the Bakelite particles horizontally (on the bed surface) and vertically (i.e., with depth) was recorded on the mudflat bed than on the saltmarsh bed, with more movement upstream (i.e., opposite to the flow direction in the annular flume) in the former as compared to the downstream sense of transport in the latter (Fig. 7).

## Discussion

### Influence of saltmarsh and mudflat on varying hydrodynamics

The steady distribution of hydrodynamic measurements on the mudflat and flatbed indicate that the flows occurred within the turbulence-dominated region of the benthic boundary layer, as described by ref.^28^. Higher than 1 cm above the saltmarsh system, turbulent forces obliterate the underlying viscous sublayer of the benthic boundary layer^28^, transmitting drag and shear stresses directly to the bed. This increase in turbulent forces within the saltmarsh and mudflat tests at this low height may be attributed to irregularities and unevenness of their bed surfaces (Fig. 4) due to benthic activities and is an indication of the effect of bed roughness on turbulent flows^29,30^^.^ The calculated hydrodynamic roughnesses, z_0_ = 10^–4^ to 10^–6^ m, were typical of muddy environments in the flatbed case^31^. A similar effect of bed roughness on the flow regimes can be observed from the mean velocities and bed shear stress (Table S2), with an increase in the downstream direction from P1 to P3.

Hydrodynamic measurements within the saltmarsh bed, up to a maximum height of 6 cm, were collected within the vegetation canopy. They show typical flow dynamics for submerged vegetation canopies as discussed in ref.^32^. Changes in the velocity and shear stress distributions observed for these test (saltmarsh) measurements, below and above the canopy, can be explained by the skimming flow effect above the canopy. Flow velocities within the canopy are strongly affected by the free flow and higher velocities above the canopy^32^. Further, Neumeier and Ciavola^18^ showed that submerged *Spartina* plants modify hydrodynamics into a low-velocity region below the canopy and a logarithmic velocity distribution above the canopy, for well-submerged vegetation hydrodynamics. This flow regime above the canopy is characterised by swift flows, bending of leaves and stems, low friction and shear stress reduction^7^. This explains the lower turbulent kinetic energy and shear stress within the saltmarsh compared to the mudflat at low motor speeds. The stress reduction effect above the canopy was reversed by the bed roughness and vegetative roughness of the saltmarsh and *Spartina* plants, respectively. This manifests in a higher bed shear stress within the saltmarsh up to a maximum of 3.3 Pa (indicative of intense turbulent momentum exchanges due to enhanced roughness) against 0.05 Pa on the mudflat.

### Erodibility and transportation of microplastics and sediments within the saltmarsh systems

The two forms of microplastics, Bakelite particles (~ 500 µm) and PVC nurdles (~ 5 mm), examined in the study showed distinct modes of transport under the increasing hydrodynamic forcing. Low velocities less than 0.053 m s^−1^ (< 15 Hz motor speed) caused negligible or no erosion of the top layer of the bed and failed to transport the PVC nurdles. This effect was greater within the saltmarsh, with the effect of the vegetative and bed roughness preventing sediment or microplastic transport. With denser vegetation and well-developed shoot systems, *Spartina* plants aid in attenuating flow velocity and enhancing sediment accretion at local scales^33^ and this translates to microplastic particles too. The same effect was observed in this experiment through reduced flow velocity (due to turbulence dissipation) and improved stabilizations by roots of the *Spartina* and filamentous biota^34^ on the saltmarsh/mudflat bed. A comparison of critical velocities for the motion of sediments in vegetated and non-vegetated beds by ref.^12^ presents lower and higher velocities for the different beds, respectively, with an emphasis on the effect of vegetation density, which may be seasonal. Comparably, a higher velocity threshold (0.25 m s^−1^) for resuspension of sediments was recorded in the mudflat bed compared to the saltmarsh where the critical velocities were below 0.0003 m s^−1^ with no significant sediment or microplastics in suspension. This further emphasizes the effect of bed roughness in increasing the thresholds for eroding the microplastics compared to the thresholds from the flatbed case (0.11 m s^−1^). Similar findings were reported by ref.^29^, in comparing drag coefficients over rough and smooth beds.

Biostabilization of the tested beds was fundamentally important to their high shear strength and resistance to erosion. In addition to the stabilization from *Spartina* roots, the formation of algal biofilms, and secretions of extracellular polymeric substances (EPS) by bacteria, *Hydrobia* snails and other organisms within saltmarsh systems are known to enhance bed shear strength (and hence resistance to erosion) by binding sediment particles^12,35,36^. These organisms (macrobenthos), together with the biofilms, are recognized as efficient eco-engineers, serving both as bioturbators and biostabilizers that shape most estuarine coastlines^27,37^. Under high flow velocity regimes (> 30 Hz motor speed/ > ~ 0.1 m s^−1^), the beds experienced Type 1a erosion (asymptotic/benign of floc/aggregates) erosion^35^, with the Bakelite particles and algal debris being eroded and resuspended. Higher flow velocities up to 0.9 m s^−1^ could achieve Type 1b erosion^35^ of fine-grained sediments comprising the surface layer of the saltmarsh bed. However, the constant SPM concentration values suggest a likely case of bed armouring^38^ occurring within the saltmarsh systems.

Higher SPM concetrations recorded for the mudflat was induced by activities of the burrowing crab and *Hydrobia* snails dwelling in the mudflat. Visual observations revealed that the feeding/grazing, burrowing, and sidling activities of the crab entrained the PVC nurdles along its path, reworked them and mobilized the sediments and Bakelites into suspension. Increased erosion of marine sediments due to the activities of *Hydrobia* snails was reported by refs.^39–41^, however, this influence from the snails was negligible in this study. This could be due to the lower population density (≤ 5 per 0.11 m^2^) compared to that reported in previous studies. In-situ estimation of the critical erosion threshold of estuarine mudflats^42^ measured with a similar annular flume by ref.^43^ yielded values between 0.75 Pa and 0.78 Pa. The corresponding erosion threshold for the 0.25 m s^−1^ critical velocity of the mudflat bed was 0.5 Pa (Table S2). This lower value obtained could be attributed to the deposition and consolidation history of our studied mudflat bed. Conversely, the estimated threshold of this study still falls within the range of erosion thresholds of mudflats discussed in ref.^44^.

For the flow velocities recorded in these experiments (≤ 0.51 m s^−1^), the PVC nurdles were scarcely in suspension. Peak tidal current velocities in Southampton Water^45^, which occur on the ebb tides and are responsible for transporting sediments seaward, are ≤ 0.59 m s^−1^. This implies that nurdle forms of microplastics within the vegetated intertidal zone are most likely to reside and stay trapped within coastal sediments when washed in by fluvial systems. Their likely resuspension or transportation further offshore is thus restricted to peak tidal currents (with velocities up to 1.0 m s^−1^) or storm conditions.

### Microplastics’ trapping and burial efficiency of the saltmarsh systems

In assessing the efficiency of saltmarshes in trapping microplastics, it is important to consider the form and size of microplastics in the saltmarsh environment. Further considerations must include the prevailing hydrodynamics, bed roughness, vegetation type and vegetation density^13^ of the saltmarsh-mudflat systems. Within the mudflat (i.e., Test C), trapping of the Bakelite particles (~ 500 µm) was achieved by their incorporation within aggregates of algal debris. Strengthening of the formed aggregates by secretions from micro-organisms in the saltmarsh is suspected, as pellets of the algal debris were formed and transported (rolling and sliding) into depressions on the bed. These aggregates provide ephemeral burial for the Bakelites and PVC nurdles, subsequently disintegrating to release the trapped microplastics at higher flow velocities or by the action of the bioturbators (crabs). The mudflat beds, alone, provided weaker trapping for the microplastics compared to the saltmarsh.

**Figure 8:**
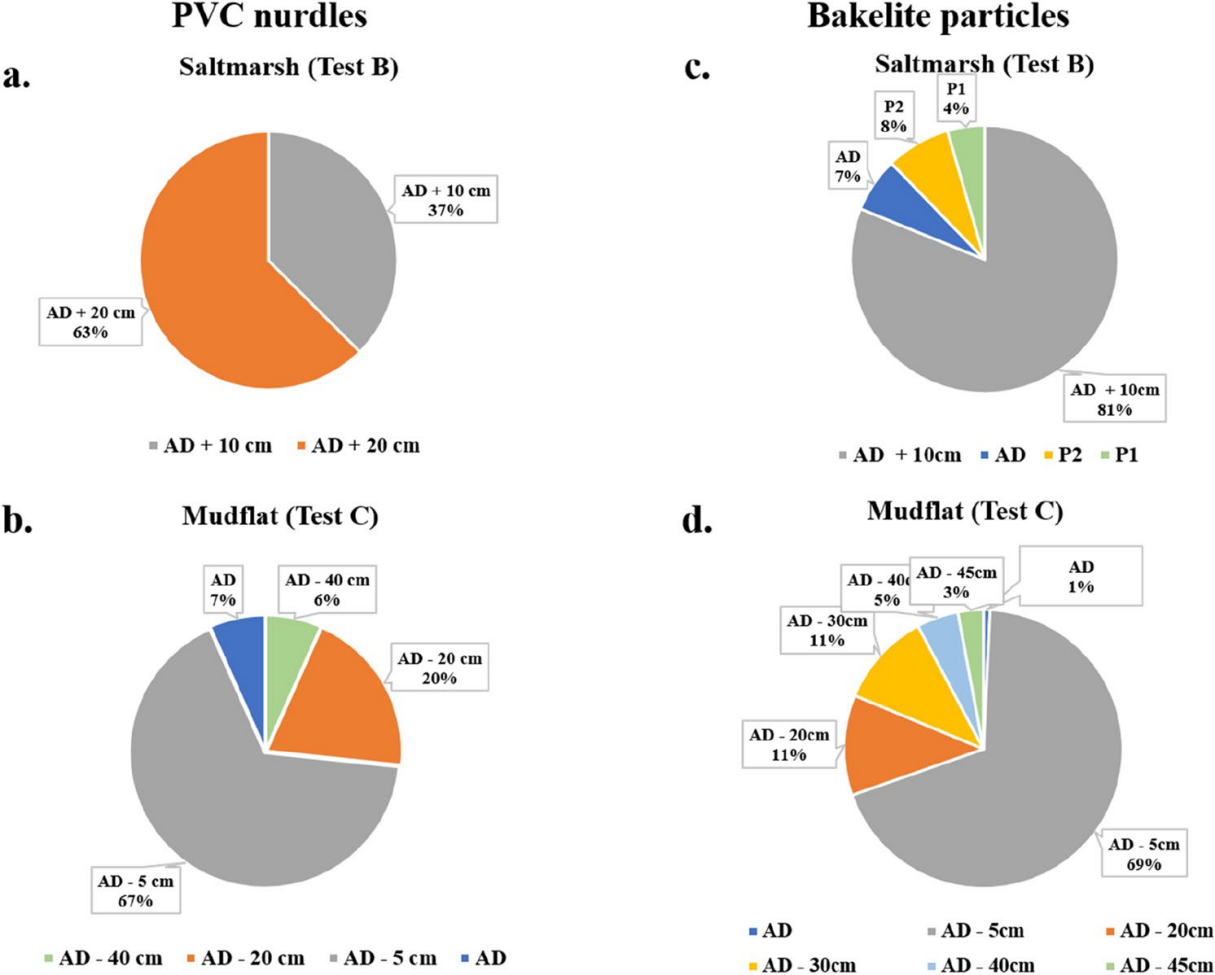
Distributions of the recovered microplastics from Figs. 6a,b and 7c,d, across the experimental tests. AD means Area of Deployment; the negative sign means upstream (against the water flow direction) and positive sign means downstream (in the direction of the water flow).

The saltmarsh bed (i.e., Test B) showed better trapping efficiency of the PVC nurdles than the mudflat and this is evidenced by the distance of transport and direction in each test/system (Fig. 8). Higher displacement of the PVC nurdles in the mudflat results from locomotion by the resident macro-organisms. The greater efficiency of the saltmarsh with *Spartina* plants in trapping the nurdle microplastics corresponds with the results of refs.^14,19^. Yao et al*.*^19^ highlighted that the microplastics’ trapping efficiency of tidal flats is dependent on bed elevation (corresponding to bed roughness in this study) and vegetation canopy. Further studies are necessary to assert the varying effects of vegetation (*Spartina* plants) density in the estuarine trapping of microplastics as ref.^46^ reported that vegetation cover and stem density have strong impacts on microplastic accumulation in wetland habitats. Applying the results of this study by utilizing saltmarsh vegetation (*Spartina* plants) in estuarine microplastics trapping, a better trapping efficiency can be achieved for flow velocities below 0.5 m s^−1^. Flows above this velocity could result in resuspension of the trapped microplastics.

The resident crab, *Carcinus maenas*, played a major role in the burial of Bakelite particles and PVC nurdles. Much impact of this bioengineering function was observed in the mudflat (Figs. 6 and 7) with samples recovered from a few cm, subsurface, in the upstream section (opposite the flow direction). Intermittent quiescent periods between the experiments were likely to have favoured the bioturbation and burial of some of the Bakelites. These quiescent periods in the experimental runs simulate standing tide periods along estuarine coasts. Crab burrows within the mudflat also served as shelters for the crabs (and microplastics) during high flow conditions as smaller crabs were observed to be swept away by the highest currents in some sections of the flume carousel. The microplastics sequestering function of crabs within the saltmarsh systems, as observed in this study, provides an additional trapping and retention mechanism for removing plastics pollution within wetlands, effectively enhancing the estuarine “filter” for microplastics. The resident crabs provided this solution by either transferring the microplastics into their trophic chain, through ingestion, from where they may end up as faecal pellets nitrifying the saltmarsh systems or through burial by their bioturbating actions as observed in this study. Similar findings on the ingestion of microplastics by copepods have been reported by refs.^47,48^. Enhancement of the estuarine “filter” through this activity may not be beneficial to the crabs themselves, as the harmful effects of microplastic ingestion and accumulation in crabs, such as changes in their feeding pattern and growth, have been highlighted by refs.^49,50^. Overall, a more efficient ‘biofiltering’ saltmarsh system for microplastics can evidently be achieved with the accumulation of flourishing halophytes for trapping the microplastics due to their hydrodynamic effect^23^, rapid sediment deposition^16^, and with infauna present (*e.g.*, the burrowing crabs) to bury the microplastics trapped by the saltmarsh halophytes.

## Conclusions

This study investigated the roles of various components of saltmarsh systems, specifically, halophytes/flora and infauna, in trapping microplastics. Using controlled flow experiments on saltmarsh and mudflat sediment monoliths in a laboratory annular flume, the conclusions and findings of this study can be summarized as follows:

1. Turbulent flow regimes, with Re > 10^5^, characterized the hydrodynamics existing within the saltmarsh systems across the flow velocities (from 0.013 to 0.51 m s^−1^) used, measured within the *Spartina* plant canopy (for the vegetated bed) and above the mudflat bed.
2. At the highest attainable velocity (0.51 m s^−1^) in the experiment, which is close to peak tidal current velocities within Southampton Water (the source of the tested sediment beds), a negligible amount of sediments and microplastics (PVC nurdles and Bakelite particles) were eroded from the saltmarsh bed. Conversely, the mudflat bed had its surface layer, consisting of algal debris and Bakelite particles, eroded at velocities greater than 0.26 m s^−1^. The *Spartina* plants increased the erosion threshold of saltmarshes as well as their trapping efficiency for microplastics. The efficiency of the algal biofilm mat on the mudflat bed in trapping Bakelite microplastics was observed under lower flow velocities (< 0.25 m s^−1^).
3. The infauna within the system, specifically the burrowing crab *Carcinus maenas*, enhanced the transportation of the denser microplastics (the PVC nurdles), the burial of both microplastic types deployed in this study, and their resuspension within the system under quiescent and turbulent flow periods. It can be concluded that their absence in the system could result in lowered transportation for the denser PVC nurdles and burial efficiency of both microplastics within the saltmarsh systems.

## Materials and methodology

### Sampling site and sample collection

**Figure 9:**
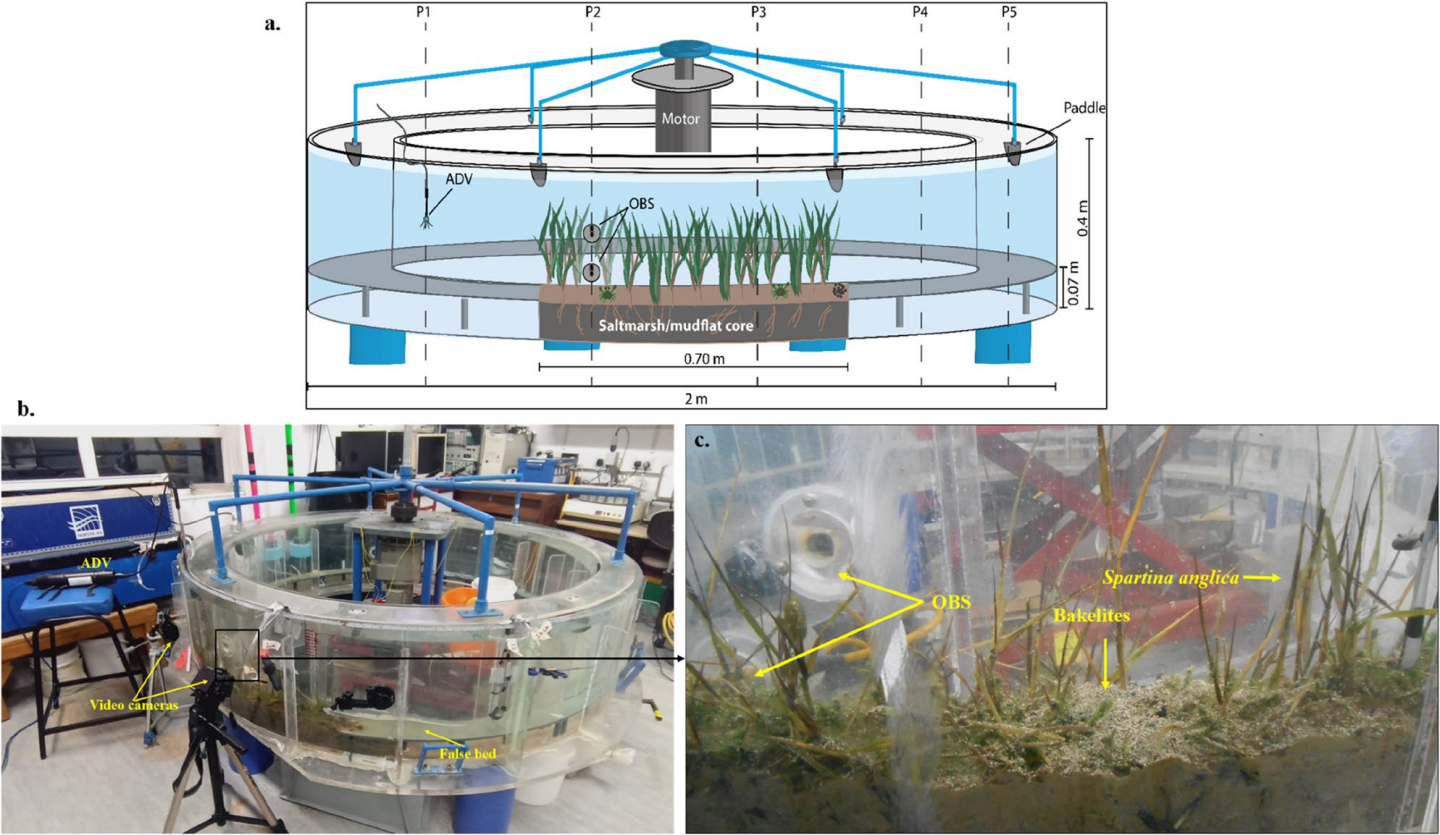
The laboratory carousel annular flume set up (**a–c**) the in-situ vegetated sediment bed experiment.

Laboratory flume experiments were conducted to assess the role of saltmarsh systems in trapping microplastics, using extracted (monolithic) cores of saltmarsh and mudflat beds from the Hythe intertidal flats zone, Southampton Water, Southern England (Fig. 1). The dominant halophyte within this saltmarsh environment is the *Spartina anglica* cordgrass species^51^. The vegetated saltmarsh surface occurs at the upper levels of the intertidal zone, while the mudflats lie on the lower reaches of the zone. Three custom-designed core boxes, each fitting one-eighth the channel circumference of the Laboratory Carousel annular flume (Fig. 9), were used to collect two saltmarsh beds bearing the *Spartina* halophyte and one bare mudflat bed. Each of the cored beds was 7 cm thick, with a surface area of 0.11m^2^. The samples with their overlying halophyte (for saltmarsh) and biofilms (for mudflat) covers were carefully transported to the laboratory to ensure that the bed structure remained relatively undisturbed.

### Experiment setup

The experiments were undertaken within a Laboratory Carousel (Fig. 9), a large acrylic annular flume modelled after the Sea Carousel^29,52–54^ at the Coastal Processes Lab, University of Southampton. The 2 m diameter annular flume has a 0.15 m channel width and 0.5 m high transparent walls, allowing direct visual observation of within-flume processes. A steady current within the carousel is generated by 8 equidistant paddles attached to a suspended lid, driven by a rotating wheel fixed to a programmable electric motor with varying motor speeds. The motor speed is computer-controlled using a calibrated frequency scale, ranging from 5 to 45 Hz. Three Optical Backscatter Sensors (OBS) were flush-mounted on the inner walls of the carousel to record the turbidity (in millivolts) at heights of 0.03 m (OBS 1; which was buried within our core, and not used hereafter), 0.09 m (OBS 2) and 0.2 m (OBS 3), above a false flume base. For these saltmarsh/mudflat tests/experiments, a false floor was constructed, flush with the core surface at 0.07 m above the flume base, to ensure a uniform flow cross-section along the channel.

Three experimental runs were performed as follows;

Test A—Flatbed clear water (for calibration)

Test B—Vegetated sediment bed (i.e., saltmarsh bearing the *Spartina anglica* cordgrass)

Test C—Flat sediment bed (i.e., mudflat)

The flume calibration (Test A) was conducted using freshwater (at 16.8 ºC; mean density of 1,000 kg m^−3^.), while tests B and C used sand-filtered seawater collected from the Southampton Water estuary (temperature 16.5–17.9 ºC; salinity 30.5–30.9 g/kg; mean water density of 1022 kg m^−3^), which aimed at maintaining the ecology of the saltmarsh systems. Test A was performed to assess the attainable flow velocities and shear stresses within the flume and determine the vertical profiles and positions for logging the velocity data. The ensuing experimental runs (tests B and C) involved successively installing the saltmarsh and mudflat sections/monoliths within the flume, with microplastics deployed at their surfaces.

**Figure 10:**
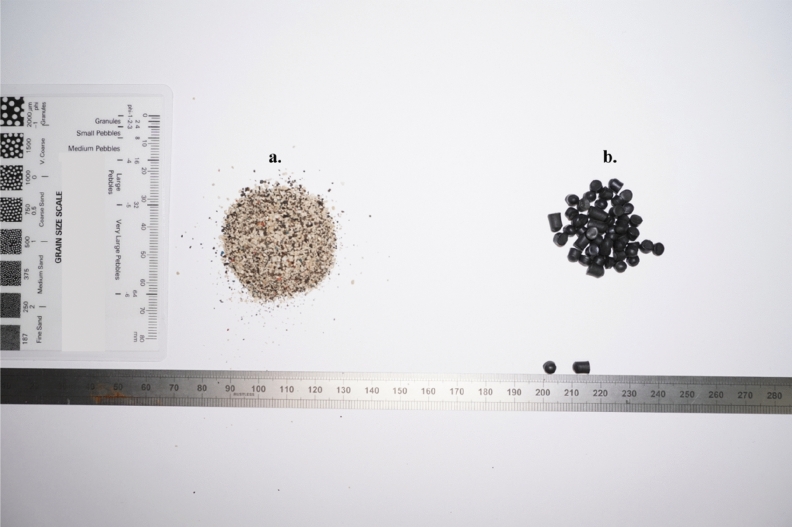
Microplastics, (**a**) Bakelite particles and (**b**) PVC nurdles, deployed in the experiment.

Two types of microplastics were deployed in the experiment, namely: Bakelite (chemically identified as polyoxybenzylmethylenglycolanhydride)^55^ particles and polyvinyl chloride (PVC) nurdles, with mean particles sizes of 500 µm and 5 mm, respectively (Fig. 10). These were selected due to their prevalence in household products and industrial goods^19^, coupled with their varying particle size and morphology, and because they occur with other microplastic forms trapped in most estuarine saltmarshes^14^. One hundred (100) g of the Bakelite particles (with a particle density of 1.3 g L^−1^) and 500 PVC nurdles (mean mass of 0.07 g per nurdle and single particle density of 1.38 g L^−1^) were placed on the surface of the saltmarsh and mudflat during each experimental run, simulating a localised spill (Fig. 9c). The threshold of incipient motion for the Bakelite particles and PVC nurdles was pre-assessed in a core mini flume (CMF) in clear water conditions by subjecting deposited beds of each of the microplastics forms to incrementally increasing flow velocities, and monitoring transport inception and suspension with the OBS and a side camera (the results are summarized in supplementary data). The core mini flume embodies a smaller model of the lab carousel with a diameter of 0.19 m, 0.34 m height and channel width of 0.04 m^56^.

### Hydrodynamics measurement, microplastics and sediment sampling methods

The Laboratory Carousel was filled with seawater to a height of 0.35 m (0.28 m above the false bed) for experimental tests B and C. Temperature and salinity of the seawater were measured to calculate its density and viscosity (See Supplementary Data). Vertical distributions of the current velocities were measured at 3 locations; profiles 1 (P1), 2 (P2) and 3 (P3) spaced 50 cm apart. Five (5) profiles were used in Test A for the calibration runs. The profiles (Fig. 9a) were carefully selected to monitor the effect of the saltmarsh systems on hydrodynamics before (upstream), within, and after (downstream) the saltmarsh systems. The motor speed was incrementally increased from 10 to 35 Hz for Test A and up to 45 Hz for tests B and C. This induced increasingly higher currents within the annular flume up to speeds sufficient to mobilise the microplastics (exceeding the threshold for motion), but not generate bed erosion.

Vertical distributions of the current velocity and turbulence at each profile were measured using a single point 10 MHz Nortek Vectrino acoustic Doppler velocimeter (ADV) manually adjusted in the vertical to construct a profile for each flowing force (Fig. 9b). The ADV measures the high-frequency 3D current velocity field (from which turbulent fluctuations can be inferred) in the streamwise (x), azimuthal (y) and vertical (z) directions, within a cylindrical sample volume (7 mm), located at 5 cm below the ADV. The velocity data were logged at a sampling rate of 25 Hz, recording 8000 data points at each height (~ 5 min at a steady state). Video cameras were strategically positioned at the beginning (P1) and middle (P2 and P3) of the saltmarsh systems to observe macroscopic processes and faunal activities within the carousel during experimental runs. Such potential activities include the transport mode of the microplastics as well as the response of the saltmarsh systems to the hydrodynamic changes. Water samples were taken with a 50 ml syringe at the end of each motor speed and filtered in a pre-ashed and pre-weighed GF/F 0.7 $\mu$*m glass fibre filter paper to determine the mass of resuspended matter (sediment and organics) and microplastics (after drying at 60 °C for 24 h) and for calibrating the OBS-recorded turbidity against the suspended particulate matter (SPM) concentration. Post-experiment syringe cores were taken from the mudflat and saltmarsh beds to estimate the quantities of buried microplastics for the two microplastic forms (Bakelite particles and PVC nurdles) and assess their extent of dispersion from the area of deployment.*

### Data analysis

The logged velocity data (binary .vno files) were recorded and processed into 2 file components (.hdr and .dat) using the Vectrino Plus software (v1.21.02). Each .hdr file contained the metadata components for the velocity measurement at each height, while the corresponding .dat files (translated values) were processed in MATLAB (R2019b, MathWorks). In the MATLAB platform, the .dat files were analysed with a set of algorithms following the methodology outlined in ref*.*^57,58^. The algorithm carefully selects the .dat files for all the heights in each profile and uses the temperature and salinity measurements to calculate the speed of sound, water density and viscosity. A quality control measure checks for signal correlation (in percentage), with the threshold for good data quality set at 70 following ref.^59^. Data points with correlation percentages below the threshold are removed and interpolated using a zero-phase, moving average algorithm. Spikes in the data (which can arise due to bubbles or wall effects) were identified using a 3D phase-space method^60^ as modified by ref.^61^, removed and then interpolated. Further despiking (likely due to reflection) was required, and this was achieved by adaptively setting quality thresholds in each time series of velocity.

The mean velocity magnitude ($\overline{u }$) for a given height is then calculated (as an ensemble average), and the fluctuations about the mean (turbulent components; ${u}^{{\prime}}, {v}^{{\prime}}$ and $w{^{\prime}}$ in the streamwise, radial and vertical directions of the flow, respectively) for each height are extracted through a Reynolds’ decomposition (${u}^{{\prime}}=u- \overline{u }$). These were used to calculate the turbulent kinetic energy (TKE or E) and to estimate the shear stress,${\tau }_{TKE}$, at each height following ref.^28^.

1 $${\tau }_{TKE}, =TKE \times 0.19$$

where 0.19 is an empirical coefficient that is constant under varying conditions^28,29,62^, and TKE is obtained from:

2 $$TKE= \frac{1}{2}\rho \left(\overline{ {u}^{{{\prime}}2}}+ \overline{{v}^{{{\prime}}2}}+ \overline{{w}^{{{\prime}}2}}\right)$$

where $\rho$ is the water density.

The flow Reynolds number (Re) was calculated to describe the state of the flow^63^ using the mean velocity for each experimental test.

3 $$Re= \frac{{U}_{Ci}L}{v}$$

${U}_{Ci}$ represents the mean velocity recorded for each experimental test (at the highest frequency); L is the water depth (0.35 m), while $v$ is the kinematic viscosity. Bed shear stress for each profile was estimated following the assumptions of the Logarithmic Law of the Wall and adopting the Von Kármán-Prandtl equation (Eq. 4) for estimating bed shear stress with multiple level velocity measurements (plot of flow velocity ${U}_{z}$ against heights, $z$ to obtain U* and z_0_ following linear equation formula, $y=mx+c$)

$${U}_{z}= \frac{{U}^{*}}{k} \mathrm{ln} \left(\frac{z}{{z}_{0}}\right)$$

4 $${U}_{z}= \frac{{U}^{*}}{k} \mathrm{ln} \left(z\right)- \frac{{U}^{*}}{k} \mathrm{ln} \left({z}_{0}\right)$$

z is the height of measurement; *U*_*z*_ is the velocity at height, z; U* is the frictional velocity; *k* is the von Kármán constant of 0.41; z_0_ is the hydrodynamic roughness length obtained from ${e}^{\frac{-c}{m}}$ (where c and m represent the intercept and slope respectively, from the linear equation plot). The bed shear stress,${ \tau }_{0}$, is therefore obtained using:

5 $${\tau }_{0}= \rho {U}^{*2}$$

The OBS data, in millivolts, were also de-spiked using a phase-space despiking algorithm and plotted against the suspended masses retained from the 50 ml water samples filtration from each test. The retained masses comprise both suspended particulates (organic and inorganic) and microplastics (Bakelite particles). Post-experiment syringe cores were dispersed in water and hydrogen peroxide. Dispersion in water aided the recovery of PVC nurdles buried during the experiment, while organic dissolution with hydrogen peroxide favoured estimating the quantity of buried Bakelite particles.

## Supplementary Information

Supplementary Information 1.

Supplementary Information 2.

Supplementary Information 3.

Supplementary Information 4.

Supplementary Information 5.

Supplementary Information 6.

Supplementary Legends.

Supplementary Video S1.

Supplementary Video S2.

Supplementary Video S3.

Supplementary Video S4.

## Data Availability

All data generated or analysed during this study are included in this published article [and its supplementary information files].
