# Supplementary material for: Role of saltmarsh systems in estuarine trapping of microplastics
Source: Sci Rep. 2022 Sep 15;12:15546. doi: 10.1038/s41598-022-18881-7 (PMC9477837; doi:10.1038/s41598-022-18881-7)
Supplement: Supplementary file 6 — Supplementary Information 6. [file 41598_2022_18881_MOESM6_ESM.docx]

## ROLE OF SALTMARSH SYSTEMS IN ESTUARINE TRAPPING OF MICROPLASTICS

## Authors and Affiliations:

## Chiedozie C. Ogbuagu^*1^, Hachem Kassem^2^, Udiba, Udiba U.^3^, Jessica L. Stead^2^, Andrew B. Cundy^2^

## ^Department of Geology, Faculty of Physical Sciences, University of Nigeria, Nsukka, 410001, Nigeria (chiedozie.ogbuagu@unn.edu.ng)^

## ^School of Ocean and Earth Sciences, National Oceanography Centre, University of Southampton, SO14 3ZH, United Kingdom (^[^Hachem.Kassem@soton.ac.uk^](mailto:Hachem.Kassem@soton.ac.uk)^;^ [^J.L.Stead@soton.ac.uk^](mailto:J.L.Stead@soton.ac.uk)^;^ [^A.Cundy@noc.soton.ac.uk^](mailto:A.Cundy@noc.soton.ac.uk)^)^

## ^Department of Zoology and Environmental Biology, University of Calabar, 540271, Nigeria (^[^udibaudiba@unical.edu.ng^](mailto:udibaudiba@unical.edu.ng)^)^

## Supplementary Information

## Hydrodynamics properties of flow in saltmarsh and mudflat

## Flatbed clear water (calibration), Test A.

The flow dynamics for this test were measured along five (5) profiles. Plots of the attained current velocities showed a constant flow distribution across the profiles, increasing steadily with increasing motor speed (Fig. 2a). The mean velocity recorded for the lowest motor speed was 0.1±0.05 ms^-1^. The highest mean velocities at 35 Hz motor speed were between 0.35 ms^-1^ and 0.39 ms^-1^ (Table 1). The TKE shear stresses plots showed similar constant shear stress distribution across the profiles (Fig. 2b). The lowest TKE shear stress calculated was 0.03 Pa at 10 Hz motor speed, while the highest motor speeds produced shear stresses of 0.26±0.02 Pa. 0.3±0.05 Pa was obtained from the highest motor speed of P2 with a mean velocity of 0.35 ms^-1^.

The Reynolds number, Re, showed the state of the flow to be turbulent (1.2 $\times$ 10^5^) at the maximum recorded velocity. Estimated z_0_ values for this experimental test were approximately zero (smooth flatbed), increasing to 10^-4^ m and 10^‑5^ m in some profiles. Similarly, values for *U** were between 10^-5^ ms^-1^ and 10^-3^ ms^-1^ yielding bed shear stresses between 10^‑5^ Pa and 0.049 Pa, respectively.

## Vegetated sediment bed (saltmarsh), Test B.

Flow dynamics over the vegetated sediment bed were measured along three (3) profiles. The distribution of the flow velocities and TKE shear stresses showed a relatively uniform distribution to a height of 4 cm above the saltmarsh bed (still within the vegetation canopy). Above this height, the flow velocities showed a typical logarithmic-shaped velocity profile (Fig. 3). For each motor speed, P3 recorded the highest mean flow velocity, from 0.032 ms^-1^ at 10 Hz to 0.32 ms^-1^ for 40 Hz (Table 2). Velocity measurements along P3 at 45 Hz were eliminated because of poor data quality. The logarithmic region of the flow (and uniform shear stress distributions) was observed to commence at a height lower than 4 cm for higher flow velocities ≥ 30 Hz. These correspond to mean flow velocities greater than 0.083 ms^-1^. Similarly, maximum TKE shear stress increased from 0.0077 Pa at 10 Hz for P3 to 3.8 Pa at 40 Hz motor speed.

The state of the flow (Re) was also turbulent (1 $\times$ 10^5^) at 0.32 ms^-1^ flow velocity. Estimated z_0_ values were between 0.0041 m at low velocities and up to 0.022 m at higher velocities. The equivalent *U** and bed shear stress were between 0.0028 ms^-1^ to 0.15 ms^-1^ and 0.008 Pa to >10 Pa, respectively.

## Flat sediment bed (mudflat), Test C.

For the flat sediment bed, constant velocity and shear stress distributions were observed across the profiles. The lowest motor speed for this test generated 0.041 ms^-1^ mean flow velocity, while 0.51 ms^-1^ was the highest mean flow velocity at 45 Hz (Table. 2). A 20 Hz motor speed was the highest attained for P1 and P2, while higher velocities were attained in P3 (Fig. 4). The lowest shear stress in each of the profiles at 10 Hz was 0.02 Pa, 0.048 Pa and 0.088 Pa for P1, P2 and P3, respectively.

The Reynolds number revealed a turbulent flow state (1.6 $\times$ 10^5^), while the hydrodynamic roughness length varied between 10^-6^ m and 10^-4^ m. Bed shear stress and *U** values were 0.00056 Pa and 0.00074 ms^-1^ at low velocities, and 0.083Pa and 0.009 ms^-1^ at higher velocities.

## Experiment conditions (more information)

Mean dynamic and kinematic viscosities for all experimental tests were approximately 0.0011 kg/ms and 1.1 * 10^-6^ m^2^s^-1^, respectively. Vegetation (stem and leaves) density of *Spartina* plants in the 2 box samples was 521 counts per 0.22 m^2^ (2368 counts per m^2^). The threshold of motion for the Bakelite particles and PVC nurdles were 0.11 ms^-1^ and 0.14 ms^-1^, respectively. The estimated mean height of the *Spartina’s* full vegetation elements (stems and leaves) was 9.5 cm and the relative vegetative roughness (RR) was computed as 0.27, following ref.^17^.

$$RR= \frac{Height of vegetation}{Height of water column} (6)$$

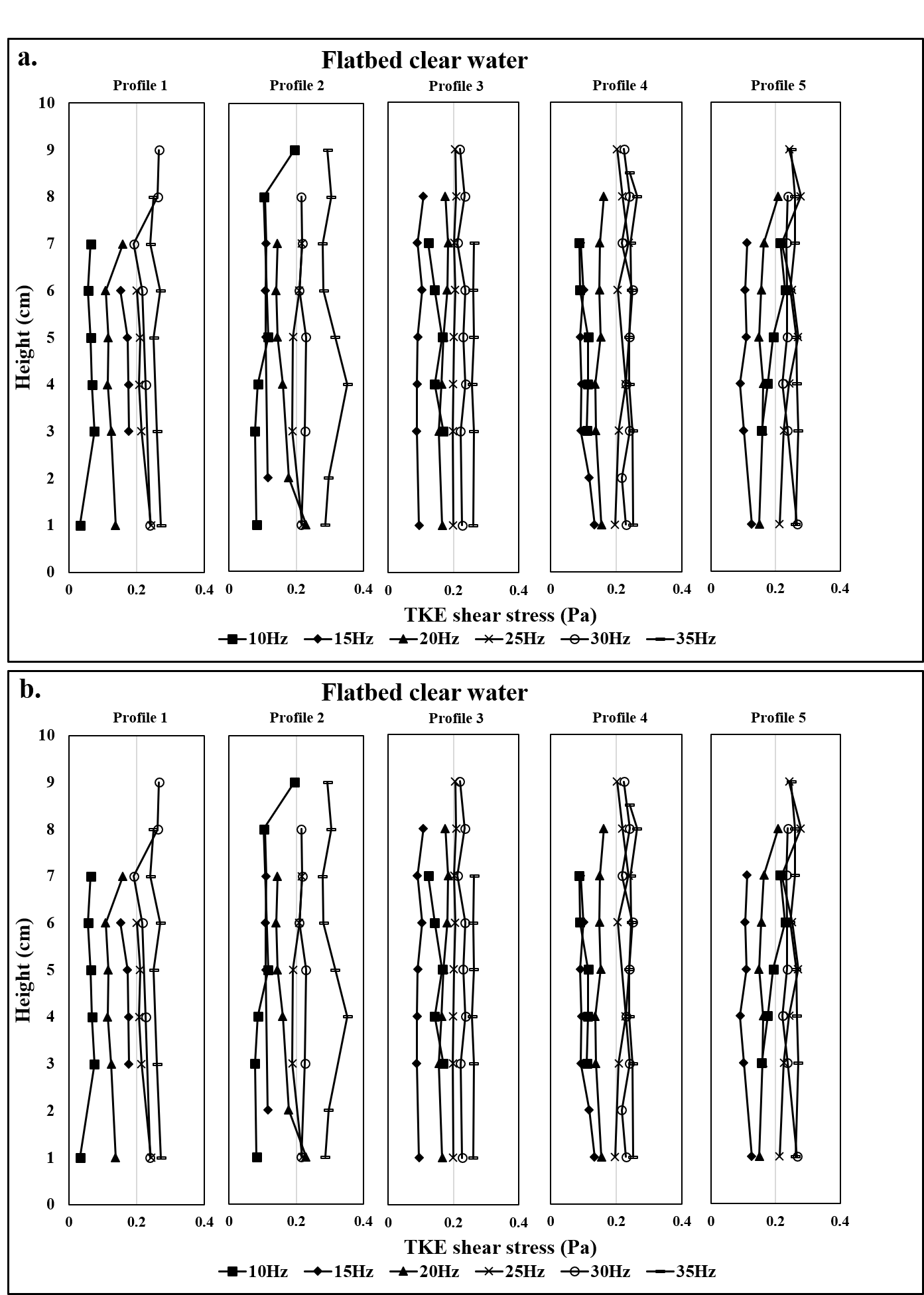
**Figure S1. Vertical distribution of (a) velocities and (b) TKE shear stresses across the profiles for flatbed clear water experiment category (Test A).**


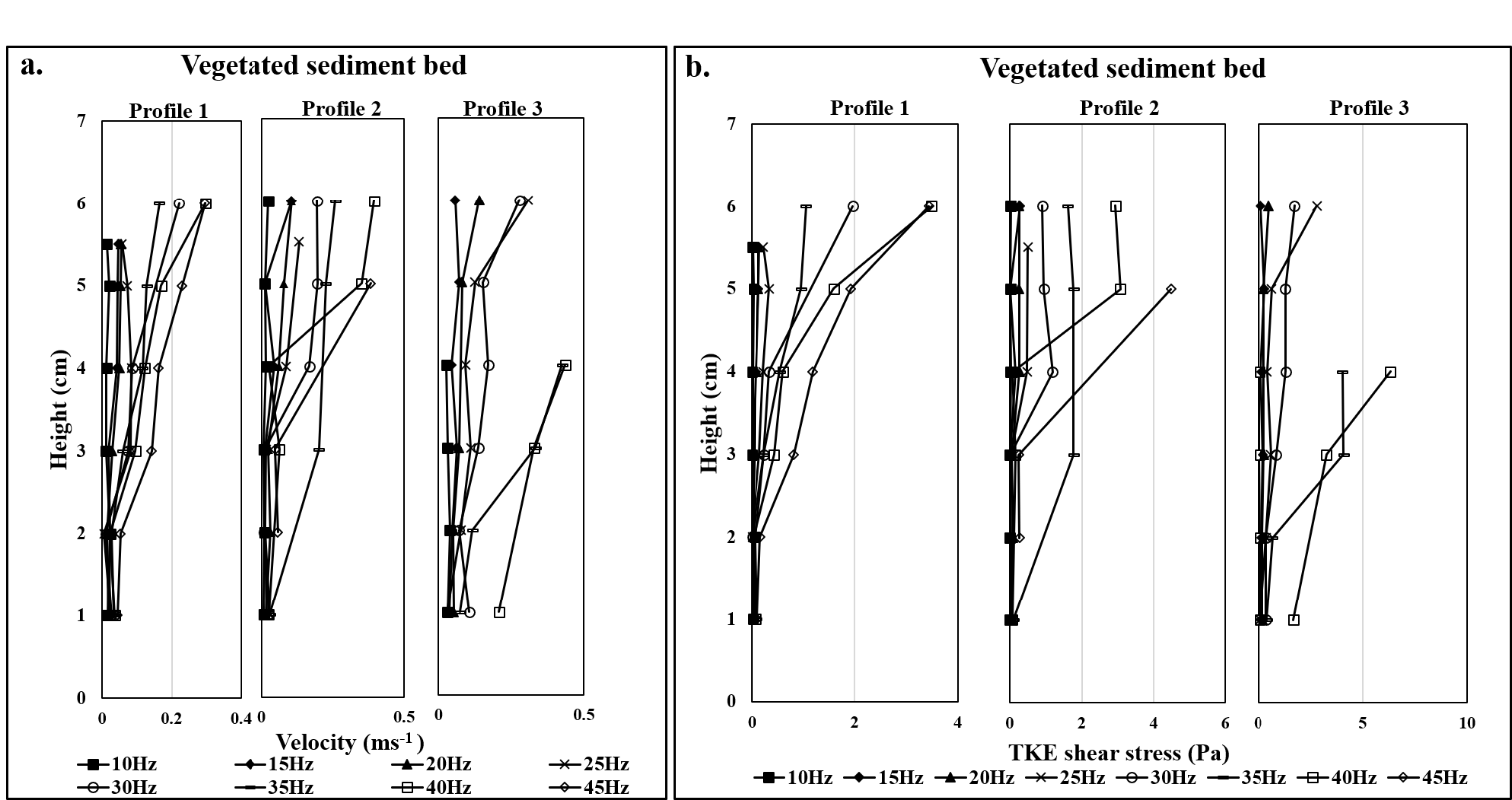


**Figure S2. Vertical distribution of (a) velocities and (b) TKE shear stresses across the profiles for vegetated sediment bed (saltmarsh with Spartina plant) experiment category (Test B).**


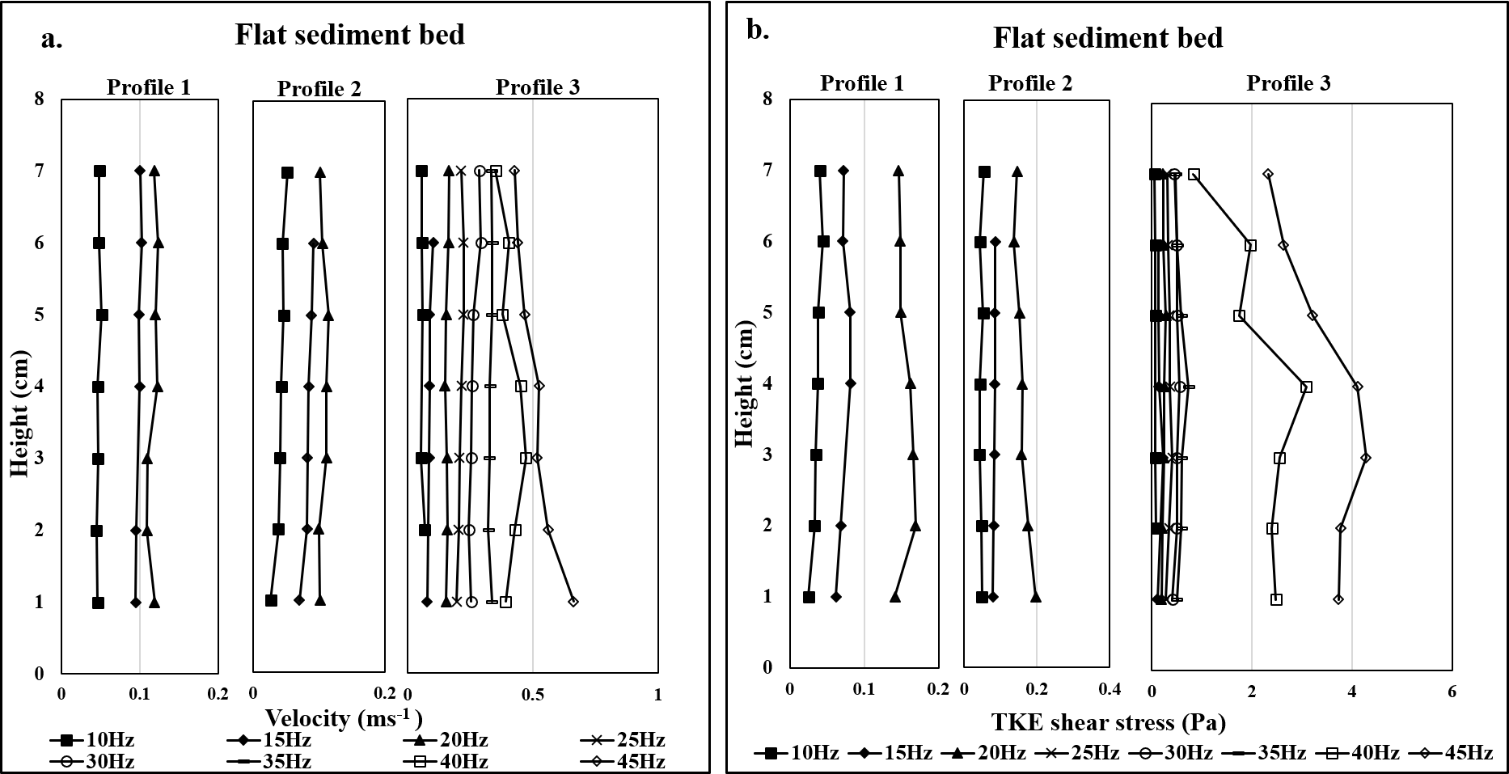
**Figure S3. Vertical distribution of (a) velocities and (b) TKE shear stresses across the profiles for flat sediment bed (mudflat) experiment category (Test C).**

**Table S1. Measured and calculated hydrodynamic parameters for the flatbed clear water experiment (Test A).**

| Flatbed clear water | | | | | | | |
| --- | --- | --- | --- | --- | --- | --- | --- |
| Motor Speed | **Profile** | **Mean Velocity (ms^-1^)** | **Bed shear stress (Pa)**  $\boldsymbol{\times}\boldsymbol{10}^{\boldsymbol{-3}}$ | **Frictional velocity, U* (ms^-1^)**  $\boldsymbol{\times}\boldsymbol{10}^{\boldsymbol{-3}}$ | **Density (kgm^-3^)** | **Z_0_ (m)**  $\boldsymbol{\times}\boldsymbol{10}^{\boldsymbol{-3}}$ | **Mean TKE shear stress (Pa)** |
| 10 Hz | 1 | 0.065 | 0.96 | 0.98 | 1000 | 0 | 0.06 |
|  | 2 | 0.075 | 38 | 6.2 | 1000 | 034 | 0.11 |
|  | 3 | 0.084 | 49 | 7 | 1000 | 0.5 | 0.15 |
|  | 4 | 0.083 | 17 | 4.1 | 1000 | 0.017 | 0.1 |
|  | 5 | 0.15 | 15 | 3.9 | 1000 | 0 | 0.19 |
| 15 Hz | 1 | 0.16 | 230 | - | 1000 | 0 | 0.17 |
|  | 2 | 0.12 | 3.2 | 0 | 1000 | 0 | 0.11 |
|  | 3 | 0.12 | 0.88 | 0.94 | 1000 | 0 | 0.094 |
|  | 4 | 0.13 | 0.96 | - | 1000 | 0 | 0.1 |
|  | 5 | 0.13 | 4 | - | 1000 | 0 | 0.11 |
| 20 Hz | 1 | 0.18 | 0.001 | 0.032 | 1000 | 0 | 0.13 |
|  | 2 | 0.17 | 0.28 | - | 1000 | 0 | 0.17 |
|  | 3 | 0.17 | 0.0067 | - | 1000 | 0 | 0.17 |
|  | 4 | 0.2 | 0.96 | 0.98 | 1000 | 0 | 0.15 |
|  | 5 | 0.2 | 1.4 | 1.2 | 1000 | 0 | 0.17 |
| 25 Hz | 1 | 0.23 | 1.7 | 1.3 | 1000 | 0 | 0.21 |
|  | 2 | 0.23 | 2.6 | 1.6 | 1000 | 0 | 0.21 |
|  | 3 | 0.24 | 2.6 | 1.6 | 1000 | 0 | 0.2 |
|  | 4 | 0.26 | 3.2 | - | 1000 | 0 | 0.21 |
|  | 5 | 0.26 | 1.4 | - | 1000 | 0 | 0.24 |
| 30 Hz | 1 | 0.29 | 0.61 | 0.78 | 1000 | 0 | 0.24 |
|  | 2 | 0.27 | 3.6 | - | 1000 | 0 | 0.22 |
|  | 3 | 0.27 | - | - | 1000 | 0 | 0.23 |
|  | 4 | 0.32 | 0.67 | - | 1000 | 0 | 0.23 |
|  | 5 | 0.32 | 5.3 | - | 1000 | 0 | 0.24 |
| 35 Hz | 1 | 0.35 | 0.24 | 0.49 | 1000 | 0 | 0.26 |
|  | 2 | 0.35 | 28 | 5.3 | 1000 | 0 | 0.3 |
|  | 3 | 0.35 | 24 | 4.9 | 1000 | 0 | 0.26 |
|  | 4 | 0.39 | 6.3 | - | 1000 | 0 | 0.25 |
|  | 5 | 0.37 | 9.6 | - | 1000 | 0 | 0.26 |

**Table S2. Measured and calculated hydrodynamic parameters for the vegetated sediment bed (Test B) and flat sediment bed (Test C).**

|  | | Vegetated sediment bed | | | | | Flat sediment bed | | | | |
| --- | --- | --- | --- | --- | --- | --- | --- | --- | --- | --- | --- |
| Motor Speed | **Profile** | **Mean Velocity (ms^-1^)** | **Bed shear stress (Pa)** | **Frictional velocity, U* (ms^-1^)**  $\boldsymbol{\times}\boldsymbol{10}^{\boldsymbol{-3}}$ | **Z_0_ (m)**  $\boldsymbol{\times}\boldsymbol{10}^{\boldsymbol{-3}}$ | **Mean TKE shear stress** | **Mean Velocity (ms^-1^)** | **Bed shear stress (Pa)**  $\boldsymbol{\times}\boldsymbol{10}^{\boldsymbol{-3}}$ | **Frictional velocity, U* (ms^-1^)**  $\boldsymbol{\times}\boldsymbol{10}^{\boldsymbol{-3}}$ | **Z_0_ (m)**  $\boldsymbol{\times}\boldsymbol{10}^{\boldsymbol{-3}}$ | **Mean TKE shear stress (Pa)** |
| 10 Hz | 1 | 0.016 | $0.45\times{10}^{-3}$ | - | 0 | 0.025 | 0.047 | 0.56 | 0.74 | 0 | 0.036 |
|  | 2 | 0.013 | $8 \times{10}^{-3}$ | 2.8 | 4.5 | 0.013 | 0.041 | 21 | 4.5 | 0.91 | 0.047 |
|  | 3 | 0.032 | $1.5 \times{10}^{-3}$ | - | 0 | 0.077 | 0.058 | 10 | - | 0 | 0.069 |
| 15 Hz | 1 | 0.032 | 0.05 | 7 | 4.1 | 0.075 | 0.098 | 2.3 | 1.5 | 0 | 0.073 |
|  | 2 | 0.035 | 0.17 | 13 | 9.1 | 0.087 | 0.083 | 21 | 4.5 | 0.017 | 0.082 |
|  | 3 | 0.053 | 0.029 | 5.3 | 0.5 | 0.18 | 0.087 | 16 | 3.9 | 0.0023 | 0.15 |
| 20 Hz | 1 | 0.036 | 0.12 | 11 | 6.7 | 0.077 | 0.12 | 2 | 1.4 | 0 | 0.16 |
|  | 2 | 0.054 | 0.37 | 19 | 9.1 | 0.16 | 0.1 | 1.5 | 1.2 | 0 | 0.16 |
|  | 3 | 0.079 | 0.26 | 16 | 4.1 | 0.3 | 0.16 | 2 | 1.4 | 0 | 0.23 |
| 25 Hz | 1 | 0.054 | 0.2 | 14 | 6.7 | 0.16 |  |  |  |  |  |
|  | 2 | 0.055 | 0.54 | 23 | 10 | 0.22 |  |  |  |  |  |
|  | 3 | 0.13 | 2.1 | 45 | 9.1 | 0.84 | 0.21 | 29 | 5.3 | 0 | 0.34 |
| 30 Hz | 1 | 0.083 | 1.7 | 41 | 12 | 0.53 |  |  |  |  |  |
|  | 2 | 0.1 | 2.5 | 49 | 14 | 0.52 |  |  |  |  |  |
|  | 3 | 0.15 | 1 | 32 | 4.5 | 1.0 | 0.26 | 83 | 9 | 0 | 0.49 |
| 35 Hz | 1 | 0.088 | 0.98 | 31 | 9.1 | 0.51 |  |  |  |  |  |
|  | 2 | 0.18 | 2.9 | 53 | 8.2 | 1.3 |  |  |  |  |  |
|  | 3 | 0.24 | 10 | 100 | 8.2 | 2.3 | 0.33 | 1.2 | 1.1 | 0 | 0.57 |
| 40 Hz | 1 | 0.12 | 2.9 | 53 | 12 | 1.0 |  |  |  |  |  |
|  | 2 | 0.17 | 5.6 | 74 | 14 | 1.3 |  |  |  |  |  |
|  | 3 | 0.32 | 3.3 | 57 | 2 | 3.8 | 0.41 | 50 | - | 0 | 2.1 |
| 45 Hz | 1 | 0.15 | 3.3 | 57 | 10 | 1.3 |  |  |  |  |  |
|  | 2 | 0.16 | 23 | 15- | 22 | 1.7 |  |  |  |  |  |
|  | 3 | - | - | - | - | - | 0.51 | 2500 | - | 0 | 3.4 |
