# Supplementary material for: Role of saltmarsh systems in estuarine trapping of microplastics
Source: Sci Rep. 2022 Sep 15;12:15546. doi: 10.1038/s41598-022-18881-7 (PMC9477837; doi:10.1038/s41598-022-18881-7)
Supplement: Supplementary file 7 — Supplementary Legends. [file 41598_2022_18881_MOESM7_ESM.docx]

**ROLE OF SALTMARSH SYSTEMS IN ESTUARINE TRAPPING OF MICROPLASTICS**

**Authors and Affiliations:**

**Chiedozie C. Ogbuagu^*1^, Hachem Kassem^2^, Udiba, Udiba U.^3^, Jessica L. Stead^2^, Andrew B. Cundy^2^**

1. **^Department of Geology, Faculty of Physical Sciences, University of Nigeria, Nsukka, 410001, Nigeria (chiedozie.ogbuagu@unn.edu.ng)^**
2. **^School of Ocean and Earth Sciences, National Oceanography Centre, University of Southampton, SO14 3ZH, United Kingdom (^**[**^Hachem.Kassem@soton.ac.uk^**](mailto:Hachem.Kassem@soton.ac.uk)**^;^** [**^J.L.Stead@soton.ac.uk^**](mailto:J.L.Stead@soton.ac.uk)**^;^** [**^A.Cundy@noc.soton.ac.uk^**](mailto:A.Cundy@noc.soton.ac.uk)**^)^**
3. **^Department of Zoology and Environmental Biology, University of Calabar, 540271, Nigeria (^**[**^udibaudiba@unical.edu.ng^**](mailto:udibaudiba@unical.edu.ng)**^)^**

**Description of Additional Supplementary Files**

1. **File name: Supplementary Video S1.mp4**
   **Description:** A video file showing the saltation (rolling and jumping) movement of the PVC nurdle. The speed of saltation is 0.003 ms^-1^.
2. **File name: Supplementary Video S2.mp4**
   **Description:** A video file showing the burrowing of sediments and burial of microplastics by the crab, *Carcinus maenas.*
3. **File name: Supplementary Video S3.mp4**
   **Description:** A video file showing bioturbation, mobilization and burial of microplastics by the crab, *Carcinus maenas.*
4. **File name: Supplementary Video S4.mp4**
   **Description:** A video file showing bioturbation, sediment softening, mobilization, erosion and burial of microplastics by the crab, *Carcinus maenas.*
5. **File name: Supplementary data.docx**
   **Description:** A word document containing detailed results of hydrodynamic regimes above the three experimental tests (tests A, B and C). This is summarized in the research paper under section 2.1 with Fig. 2, and in ‘Supplementary data’ document with Figs S1 to S3; Tables 1 and 2.
6. **File name: Supplementary Data 1_ADV_velocities and Shear stresses.xlsx**
   **Description:** An excel file containing all the raw parameters plotted in Fig 2; the processed ADV measured velocities, calculated TKE shear stresses, bed shear stress, frictional shear stress ($U^{*}$), hydrodynamic roughness length (z_0_) and water density for all the profiles, motor frequencies and heights measured in the experiments.
7. **File name: Supplementary Data 2_ADV_processed_Density _Viscosity.xlsx**
   **Description:** An excel file containing the calculated water densities, dynamic and kinematic viscosities at various heights and profiles for all experimental runs.
8. **File name: Supplementary Data 3_Bakelite_burial recovery.xlsx**
   **Description:** An excel file containing the mass of buried Bakelite particles per cm depth, plotted in Fig. 7.
9. **File name: Supplementary Data 4_PVC nurdle_burial recovery.xlsx**
   **Description:** An excel file containing the mass of buried PVC nurdles per cm depth, plotted in Fig. 6.
10. **File name: Supplementary Data 5_OBS_SSC_Time_series.xlsx**
    **Description:** An excel file containing the OBS and SSC raw data plotted in Fig. 3. The OBS and SSC values change with increasing motor frequency (Hz).
